# 6/m – Noch nicht so sattelfest: Vorbereitung auf die Facharztprüfung: Fall 66

**DOI:** 10.1007/s00113-021-00963-2

**Published:** 2021-02-23

**Authors:** Annelie-Martina Weinberg, Christoph Röder

**Affiliations:** 1 Universitätsklinik für Orthopädie und Unfallchirurgie, MUG Graz, Auenbruggerplatz 5, 8034 Graz, Österreich; 2 Abteilung für Orthopädie & Traumatologie, Landesklinikum Baden-Mödling, Standort Mödling, Sr. M. Restituta-Gasse 12, 2340 Mödling, Österreich; 3 Donau-Universität Krems, Dr.-Karl-Dorrek-Str. 30, 3500 Krems, Österreich

**Keywords:** Unterarmverletzungen, Diaphyse, Intramedulläre Nagelung, Refraktur, Grünholzfraktur

## Prüfungssimulation

### Fallschilderung

Ein 6‑jähriger Junge wird nach einem Sturz vom Fahrrad in die Notfallaufnahme eingeliefert. Er gibt auf Fragen Schmerzen am linken Unterarm an, ist weinerlich, und eine Anamnese kann nur schwer erhoben werden. Die Extremität ist von den Sanitätern auf einer Schiene ruhiggestellt.

## Prüfungsfragen

- Worauf ist bei der klinischen Untersuchung besonders zu achten?
- Welche apparativ–diagnostischen Verfahren sind anzuwenden?
- Nennen Sie die Einteilung der kindlichen Frakturen am Unterarm.
- Welche biomechanische Besonderheit muss bei der Behandlung von Unterarmschaftfrakturen beachtet werden?
- Welche typischen Besonderheiten kennzeichnen die Frakturheilung im Kindesalter?
- Nennen Sie die gängigen Versorgungskonzepte für die diaphysäre Unterarmschaftfraktur.
- Nennen Sie die möglichen Komplikationen und deren Vermeidung.

### Antworten

#### Worauf ist bei der klinischen Untersuchung besonders zu achten?

- Neurologischer Status: Ausschluss von Schädel- und Kopfverletzungen,
- abdominelle Verletzungen durch den Fahrradlenker (häufigster Läsionsort ist die Milz), Inspektion des gesamten Körpers mit Schwerpunkt auf dem Abdomen (z. B. Hämatome im Bauchbereich), ggf. Ultraschalluntersuchung,
- Status der Fraktur: geschlossen oder offen,
- bei offenen Frakturen II. und III. Grades: Überprüfung des Tetanusschutzes, Antibiotikaprophylaxe,
- Allergien.

##### Der Fall.

Die allgemeine Untersuchung hat keine zusätzlichen Verletzungen gezeigt. Abdominelle Sonographie, Blut- und Urinuntersuchungen ergeben unauffällige Befunde. Die periphere Durchblutung, Motorik und Sensibilität des linken Unterarms sind intakt. Es handelt sich um eine geschlossene Fraktur; Allergien liegen nicht vor.

#### Welche apparativ–diagnostischen Verfahren sind anzuwenden?

**Figure 1:**
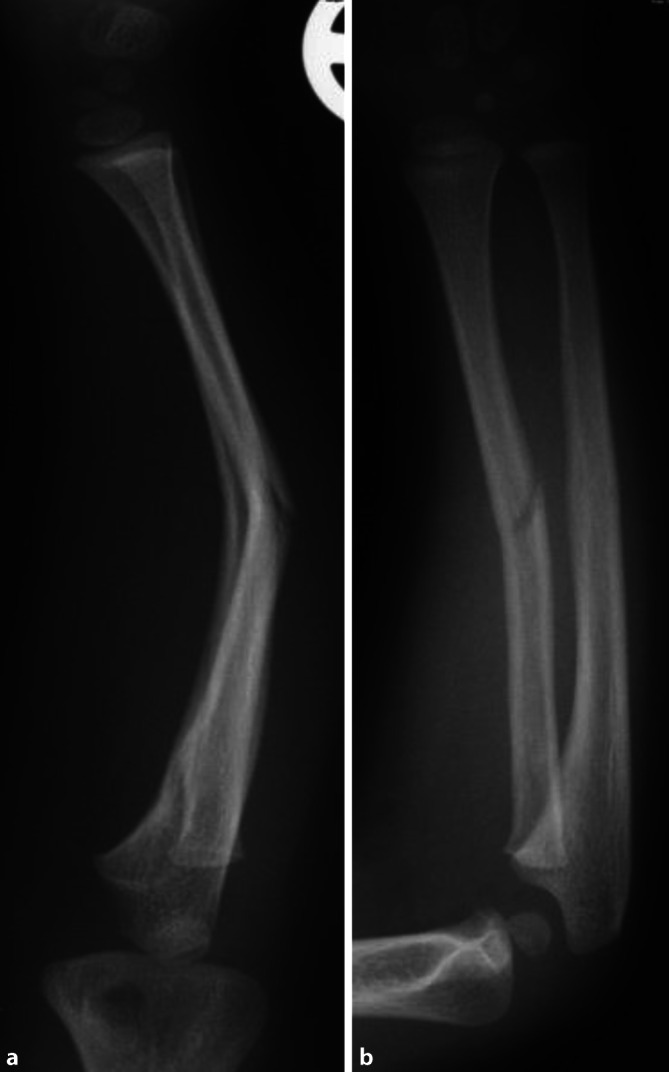

- Die Standardröntgendiagnostik ist eine Aufnahme des Unterarms mit angrenzenden Gelenken in 2 Ebenen (Abb. 1). Es muss darauf geachtet werden, dass die Aufnahmen in 2 senkrecht zueinander stehenden Ebenen durchgeführt werden, sonst kann die tatsächliche Fehlstellung unentdeckt oder fälschlicherweise als zu gering angenommen werden. Gelingt dies nicht, muss die Röntgenröhre entsprechend geschwenkt werden.

##### Der Fall.

Es handelt sich um eine geschlossene diaphysäre Unterarmschaftfraktur.

#### Nennen Sie die Einteilung der kindlichen Frakturen am Unterarm

Grundsätzlich werden kindliche Frakturen des Unterarmschaftes in distale (epi- und metaphysäre Frakturen), mittlere (diaphysäre) und proximale (meta- und epiphysäre) Frakturen eingeteilt. Das Quadrat über der Wachstumsfuge ist in den meisten Frakturklassifikationen als Metaphyse definiert. Anatomisch gesehen ist die Fuge die becherförmige Aufweitung der Diaphyse.

Aus der Einteilung in epi-, meta- und diaphysäre Frakturen können Therapiealgorithmen abgeleitet werden.

- Epiphysäre Frakturen sind anders als die Epiphysiolyse eine Rarität im Kindesalter und werden nach Salter und Harris als Typ-III- und Typ-IV-Frakturen klassifiziert.
- Metaphysäre Frakturen werden eingeteilt in:
  - Epiphysenlösung mit oder ohne metaphysären Keil (Typen I und II nach Salter und Harris),
  - vollständige Frakturen,
  - Grünholz‑/Wulstfrakturen.
- Die diaphysären Frakturen beinhalten:
  - vollständige Frakturen,
  - Grünholzfrakturen,
  - „bowing fractures“ (Biegungsfrakturen) mit oder ohne Infraktion. Unter Infraktion werden kleine Einrisse in der Kortikalis verstanden, die im radiologischen Bild oft nicht zu sehen sind.

##### Der Fall.

Die Fraktur lässt sich als diaphysäre Grünholzfraktur des Radius und Bowing fracture der Ulna klassifizieren. Dabei ist zu beachten, dass Grünholzfrakturen – zu denen auch die Biegungsfrakturen zu zählen sind –im Verlauf der Heilung eine knöcherne Durchbauungsstörung auf der konvexen Seite aufweisen; dies führt zu einer erhöhten Rate von Refrakturen an der Diaphyse. In der Metaphyse, die am Radius sehr nahe der hochpotenten Fuge lokalisiert ist, finden sich deutlich weniger Refrakturen. Man geht davon aus, dass hier die Heilung wesentlich schneller verläuft.

#### Welche biomechanische Besonderheit muss bei der Behandlung von Unterarmschaftfrakturen beachtet werden?

Der Unterarm verfügt über eine biomechanische Besonderheit durch die Umwendbewegung, die mit einem Überkreuzen der Knochen einhergeht. Distal der Überkreuzung beider Knochen ist die Funktion per se nie eingeschränkt, da sich die Knochen voneinander wegbewegen – auch wenn keine vollständige spontane Korrektur einer Fehlstellung eintritt. Proximal der Überkreuzung beider Knochen können – manchmal auch kleinste Fehlstellungen – zu Bewegungseinschränkungen der Pro- und Supination führen [6, 16, 17]. Dies sind v. a. folgende Achsfehlstellungen:

- Im a.-p.-Röntgenbild: Fehlstellungen, die den interossären Raum verengen (radiale Fehlstellungen der Ulna sowie ulnare Fehlstellungen des Radius),
- im seitlichen Röntgenbild: volare Fehlstellungen des Radius sowie dorsale Fehlstellungen der Ulna.

##### Der Fall.

**Figure 2:**
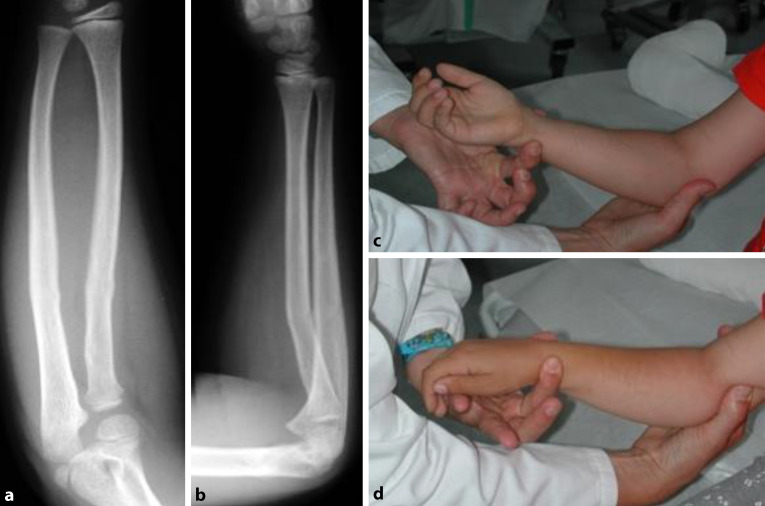

Die radiologischen Aufnahmen zeigen keine Einengung des interossären Raumes im a.-p.-Röntgenbild sowie in der seitlichen Ebene eine dorsale Achsabweichung von ca. 5–10°. Sollte die Achsfehlstellung nur schwer zu beurteilen sein (z. B. wegen Gipsüberlagerung), gehen die Autoren wie folgt vor: Bei stabilen Frakturen, deren mögliche Achsabweichung zu einer Einschränkung der Umwendbewegung führen können und die initial einer konservativer Behandlung zugeführt wurden, ist spätestens nach 7 bis 10 Tagen eine Funktionsprüfung vorzunehmen: Können aus der Neutralnullstellung heraus mühelos eine schmerzfreie Pronation und Supination von mindestens 40° erreicht werden, ist eine freie Funktion zu erwarten. Ein Beispiel zeigt Abb. 2.

#### Welche typischen Besonderheiten kennzeichnen die Frakturheilung im Kindesalter?

- Die **stimulative Wachstumsstörung** ist dadurch gekennzeichnet, dass diese nach jeder Fraktur obligat auftritt und vom Ausmaß des Remodeling der Fraktur und vom Wachstumspotenzial des betroffenen Knochens abhängt. Somit wird die Fuge zum Mehrwachstum angeregt, bis kein stattgehabter Bruch mehr im Knochen zu sehen und der Knochen vollständig wiederhergestellt ist. Damit dauert dieser Prozess sehr viel länger als die Frakturheilung per se. Dies kann zu Alterationen der Länge und in der Konsequenz am Unterarm zu Minus- bzw. Plusvarianten des Unterarms führen. Dieses Risiko ist im Bereich des Unterarmschaftes gering, da der Partnerknochen auch Längendifferenzen im Wachstum wieder ausgleichen kann. Klinische Relevanz hat dagegen die Beinlängendifferenz als stimulative Wachstumsstörung an der unteren Extremität [4].
- Die **hemmende Wachstumsstörung** führt zu einem teilweisen oder völligen Verschluss der Fuge. Diese tritt an der oberen Extremität im Gegensatz zur unteren Extremität kaum auf. Aber es kann z. B. nach einer Infektion oder Verletzungen der distalen Radiusfuge durch unsachgemäßes Einbringen der intramedullären Marknägel oder auch Manipulationen etwa durch häufiges Bohren von Kirschner-Drähten zum Verschluss der distalen Radiusepiphyse kommen, was klinisch zur Deviation der Achse und/oder zu einem kompletten Wachstumsstopp am Radius führen kann und im Bereich der Ulna eine Plusvariante verursacht. Die Patienten kommen erst verspätet bei Beschwerden in das Krankenhaus, da die klinische Relevanz verzögert eintritt [4].
- **Spontankorrekturen**. Grundsätzlich sind diese von der Frakturlokalisation, den angrenzenden Fugen und deren Wachstumspotenzial sowie vom Alter des Kindes abhängig. Am Unterarm gehen 80 % des Wachstums von der distalen Radiusepiphyse aus und nur 20 % vom Ellenbogen. Generell gilt, dass die Korrekturen in der Bewegungsebene (Sagittalebene) besser erfolgen als in der Frontalebene. Je näher die Fraktur an der Fuge gelegen und je größer das Potenzial der Fuge ist, desto mehr besteht die Fähigkeit des spontanen Remodeling. Diese Prinzipien können in ein Behandlungskonzept durch Beachtung der jeweiligen tolerablen Grenzen integriert oder als Entscheidungshilfe bei „second opinion“ nach fehlverheilten oder in Heilung befindlichen Frakturen einbezogen werden. Am Unterarmschaft beträgt das Korrekturpotenzial zwischen 5 und 15°, wobei dies stark altersabhängig ist und in der Literatur die Angaben dazu schwanken. Prospektive, die Fragestellung beantwortende Studien fehlen dazu noch. Des Weiteren korrigieren sich Fehlstellungen, die biomechanisch relevant sind, nicht. Dies bedeutet bereits bei der initialen Therapie, dass keine funktionseinschränkende Fehlstellung belassen werden darf [6].

##### Der Fall.

Die Fraktur weist eine dorsale Achsabweichung von ca. 5–10° auf, die im weiteren Wachstum bei einem Alter von 6 Jahren korrigiert werden kann. Selbst wenn diese bestehen bleibt und nur in geringerem Ausmaß ausgeglichen wird, wird sie keine Einschränkung der Umwendbewegung verursachen und keine kosmetische Alteration nach sich ziehen.

#### Nennen Sie die gängigen Versorgungskonzepte für die diaphysäre Unterarmschaftfraktur.

Am Anfang steht die Überlegung, ob es sich um eine **stabile **oder **instabile **Fraktur handelt. Als stabil gelten alle nichtvollständigen [1, 4, 11] Frakturen und solche, die nach einer Reposition stabil bleiben, wenn die Kortikalis weiterhin intakt ist.

Des Weiteren muss geprüft werden, ob die Reposition einer Narkose bedarf.

In Narkose sollte immer eine definitive Behandlung (Osteosynthese) angestrebt werden, zur Vermeidung von sekundären Dislokationen und sekundären Komplikationen wie Refrakturen bei Grünholzfrakturen [13, 14, 18].

- Konservative Therapie mit Oberarmgips [1, 4, 15]:
  - Stabile Frakturen, wenn sie nicht eine Einschränkung der Funktion nach sich ziehen (*Cave*: proximale Fehlstellungen nach Überkreuzung der Knochen beachten) und keine Dislokation aufweisen, die altersentsprechend im Verlauf nicht remodelliert werden kann (max. 10–15° [1, 4, 17]).
    Vollständige Frakturen ohne Dislokation. Hier müssen aber die Eltern aufgeklärt werden, dass ein hohes Potenzial zur sekundären Dislokation besteht. Daher werden diese Frakturen in deutschsprachigen Ländern oft primär fixiert, v. a., wenn die Nachbehandlung nicht in einer Hand bleibt.
- Indikationen zur Reposition mit nachfolgender Osteosynthese [1, 2]:
  - Frakturen im proximalen Unterarmschaft (s. Abschn. „Welche biomechanische Besonderheit muss bei der Behandlung von Unterarmschaftfrakturen beachtet werden?“ und „Welche typischen Besonderheiten kennzeichnen die Frakturheilung im Kindesalter?“).
  - Dislozierte komplette Unterarmschaftfrakturen.
  - Vollständige Schaftfraktur von Radius oder Ulna in Kombination mit einer Grünholzfraktur des jeweiligen anderen Unterarmknochens, wenn eine Narkose zur Reposition benötigt wird. (Der iatrogene Bruch der Gegenkortikalis auf der Seite der Grünholzfraktur ist Bestandteil des Therapiekonzepts.)
  - Dislozierte Grünholzfrakturen von Radius oder Ulna, wenn das Ausmaß der Dislokation einer Narkose zur Reposition bedarf.
  - Offene Frakturen der Grade II und III.
  - Frakturen im Rahmen von Mehrfachverletzungen, Polytraumen.

Das gängige Osteosyntheseverfahren ist die intramedulläre flexible Markdrahtung (Abkürzungen und auch Handelsnamen sind: TEN/STEN [elastisches Nagelsystem aus Titan/Stahl]; FIN [„flexible intramedullary nailing“], ESMN [elastisch stabile Marknagelung], ESIN [elastisch stabile intramedulläre Nagelung]). Plattenosteosynthesen kommen nur in Ausnahmefällen und eher in der Adoleszenz bei nahezu geschlossenen Fugen vor, oder selten bei Stückfrakturen, die nicht „aufgefädelt“ werden können [3]. Grundsätzlich wird die Fixation nur eines Knochens nicht empfohlen, da keine funktionelle Nachbehandlung durchgeführt werden kann.

##### Der Fall.

**Figure 3:**
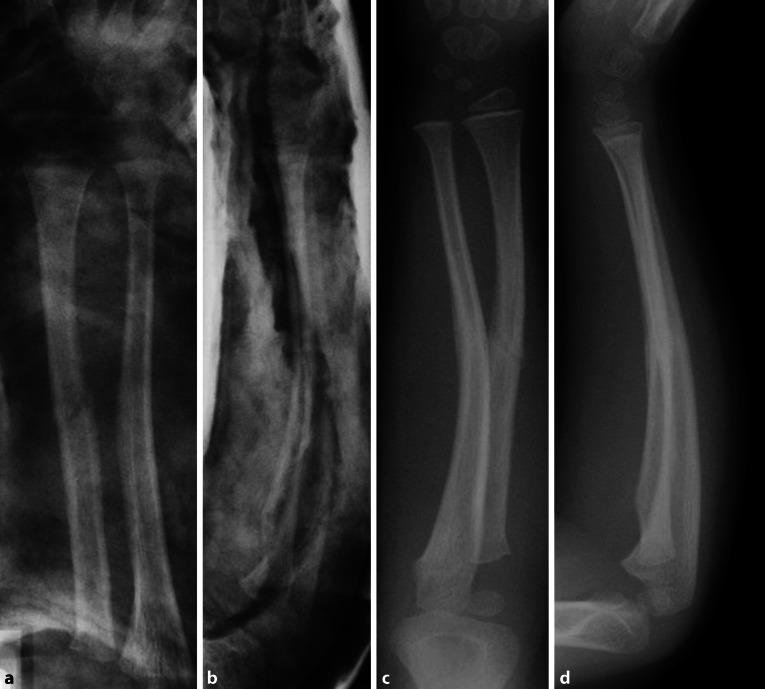

Definitionsgemäß handelte es sich um eine stabile Fraktur, die nach Aushang in Analgosedierung konservativ weiterbehandelt wurde. Die Umwendbewegung zeigte am 8. Tag nach Trauma keine Einschränkung (Abb. 3) Alternativ hätte diese Fraktur primär operativ stabilisiert werden können.

#### Nennen Sie die möglichen Komplikationen und deren Vermeidung.

- Komplikationen der konservativen Therapie [15]:
  - bei nicht vollständig durchbrochenen Grünholzfrakturen kann es nach konservativer Behandlung in bis zu 16 % der Fälle zu Refrakturen kommen [7, 18];
  - sekundäre Achsabweichungen bei kompletten Unterarmfrakturen;
  - Einschränkungen der Umwendbewegung bei Frakturen proximal der Überkreuzungsstelle beider Knochen und Achsabweichungen über 5–10°.
- Operative Therapie [5, 8, 10, 12]:
  - Refraktur nach zu früher Entfernung des Osteosynthesematerials [9];
  - Läsion des R. superficialis des N. radialis dorsalis an der distalen Eintrittsstelle des Nagels;
  - Läsion des N. ulnaris an der proximalen Eintrittsstelle des Nagels;
  - Pseudobursitis bei zu weit herausstehenden Nägeln an der proximalen Ulna;
  - hemmende Wachstumsstörungen bei unsachgemäßer operativer Technik (Verletzung der Wachstumsfuge durch falschen Eintrittspunkt des Nagels oder mehrfache Bohrversuche).

##### Der Fall.

**Figure 4:**
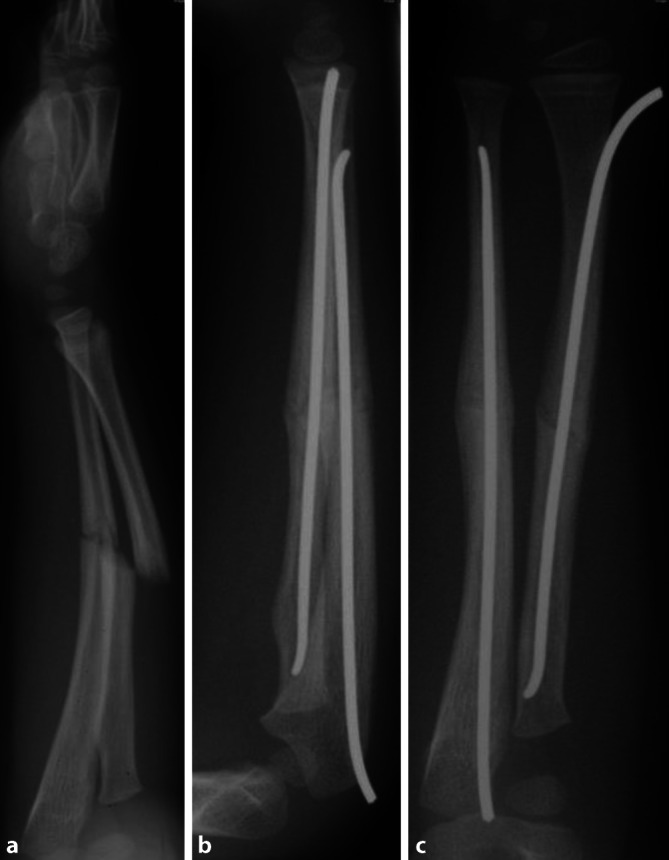

Trotz regelrecht durchgeführter konservativer Therapie kam es 3 Monate nach dem Trauma zur Refraktur. Am darauffolgenden Tag wurde die Refraktur mithilfe des ESIN-Verfahrens osteosynthetisch versorgt (Abb. 4). Der Junge konnte 4 Monate nach der Osteosynthese eine freie Umwendbewegung ausführen und wies eine konsolidierte Fraktur auf, die sich im Remodeling befand.

## References

[1] AMWF Leitlinien

[2] Dietz HG, Schmittenbecher PP, Slongo T, Wilkins KE (2006). Elastic stable intramedullary nailing (ESIN) in children—AO Manual of fracture management.

[3] Fernandez FF, Egenolf M, Carsten C (2005). Unstable diaphyseal fractures of both bones of the forearm in children: plate fixation versus intramedullary nailing. Injury.

[4] v Laer L, Kraus R, Linhart WE (2013). Frakturen und Luxationen im Wachstumsalter.

[5] Lieber J, Joeris A, Knorr P (2005). ESIN in forearm fractures: clear indications, often used, but some avoidable complications. Eur J Trauma.

[6] Loegmakers JW, Verheyen CCPM (2006). Acceptance of angulation in the non-operative treatment of paediatric forearm fractures. J Pediatr Orthop B.

[7] Makki D, Kheiran A, Gadiyar R, Ricketts D (2014). Refractures following removal of plates and elastic nails from paediatric forearms. J Pediatr Orthop B.

[8] Martus JE, Preston RK, Schoenecker JG, Lovejoy SA, Green NE, Mencio GA (2013). Complications and outcomes of diaphyseal forearm fracture intramedullary nailing: a comparison of pediatric and adolescent age groups. J Pediatr Orthop.

[9] Rousset M, Mansour M, Samba A, Pereira B, Canavese F (2016). Risk factors for re-fracture in children with diaphyseal fracture of the forearm treated with elastic stable intramedullary nailing. Eur J Orthop Surg Traumatol.

[10] Peterlein CD, Modzel T, Hagen L, Ruchholtz S, Krüger A (2019). Long-term results of elastic-stable intramedullary nailing (ESIN) of diaphyseal forearm fractures in children. Medicine.

[11] Schmittenbecher PP (2005). State-of-the-art treatment of forearm shaft fractures Injury. Int J Care Inj.

[12] Schmittenbecher PP, Dietz HG, Linhart WE (2000). Complications and problems in intramedullary nailing of children’s fractures. Eur J Trauma.

[13] Schmuck T, Altermatt S, Büchler P (2010). Greenstick fractures of the middle third of the forearm. A prospective multi-centre study. Eur J Pediatr Surg.

[14] Schwarz AF, Höcker K, Schwarz N, Jelen M, Styhler W, Mayr J, Brass D, Jansky W, Poigenfürst J, Straub G (1996). Recurrent fractures of the pediatric forearm. Unfallchirurg.

[15] Sinikumpu JJ, Victorzon S, Antila E, Pokka T, Serlo W (2014). Nonoperatively treated forearm shaft fractures in children show good long-term recovery. Acta Orthop.

[16] Vittas D, Larsen E, Torp-Pedersen S (1991). Angular remodeling of midshaft forearm fractures in children. Clin Orthop Relat Res.

[17] Weinberg A-M, Kasten P, Castellani C (2001). Which axial deviation results in limitations of pro- and supination following diaphyseal lower arm fractures in children. Eur J Trauma.

[18] Weinberg AM, Amerstorfer F, Fischerauer EE, Pearce S, Schmidt B (2009). Paediatric diaphyseal forearm refractures after greenstick fractures: operative management with ESIN. Injury.

[19] Weinberg AM, Altermatt S, Hell A, Reilmann H, Weinberg AM, Tscherne H (2006). Unterarm. Unfallchirurgie im Kindesalter (Allgemeiner Teil Kopf Obere Extremität Wirbelsäule).

